# Supplementary figures and images for: Nutritional properties of selected superfood extracts and their potential health benefits
Source: PeerJ. 2021 Nov 26;9:e12525. doi: 10.7717/peerj.12525 (PMC8628624; doi:10.7717/peerj.12525)

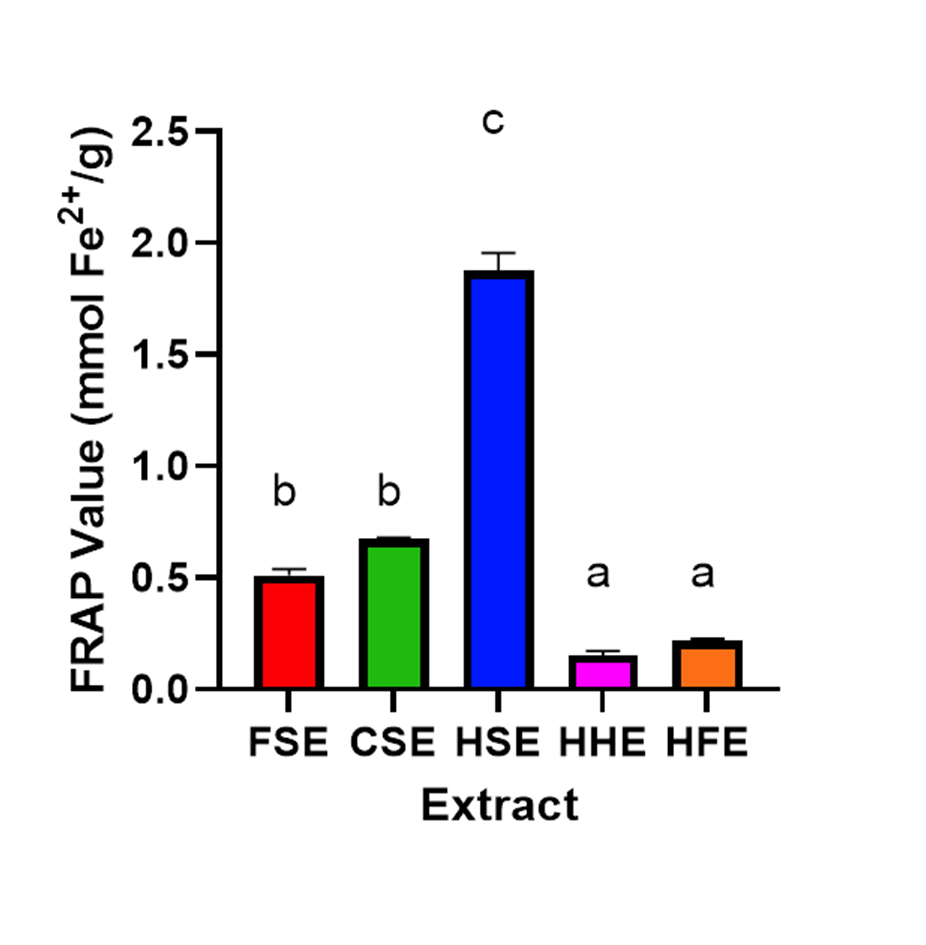

Supplement: Supplemental Information 1 — Tukey’s HSD was performed to determine statistical significance between samples (P < 0.05). [file peerj-09-12525-s001.png]

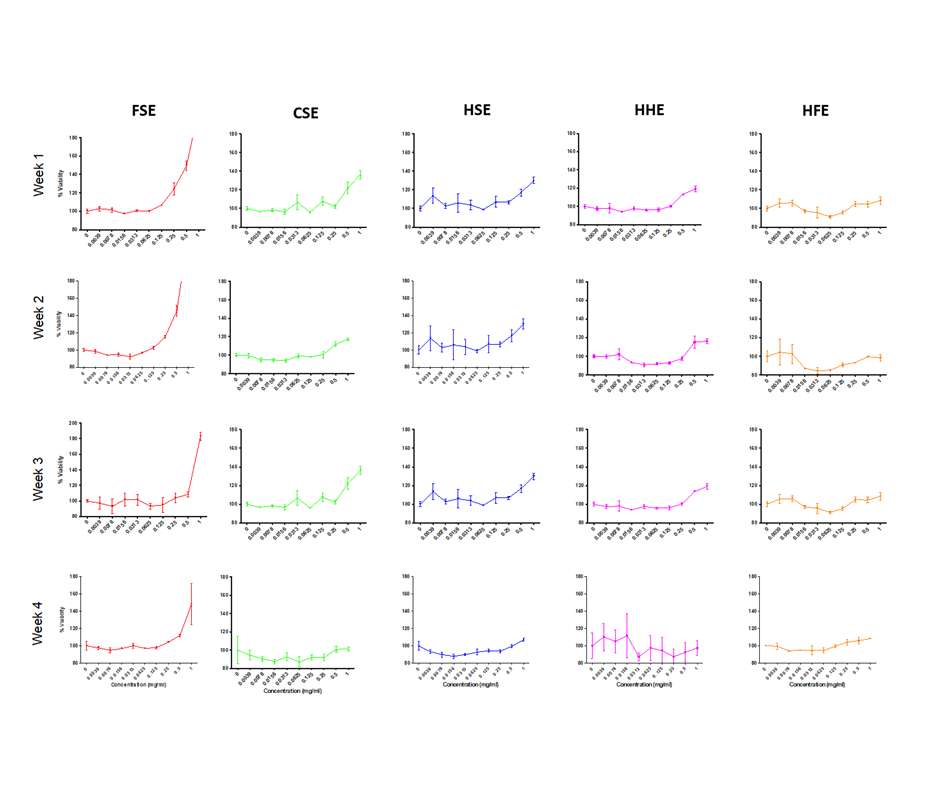

Supplement: Supplemental Information 2 — Treatments were performed in triplicate per concentration and experiments were performed on four different passages of cells over 4 separate weeks. [file peerj-09-12525-s002.png]

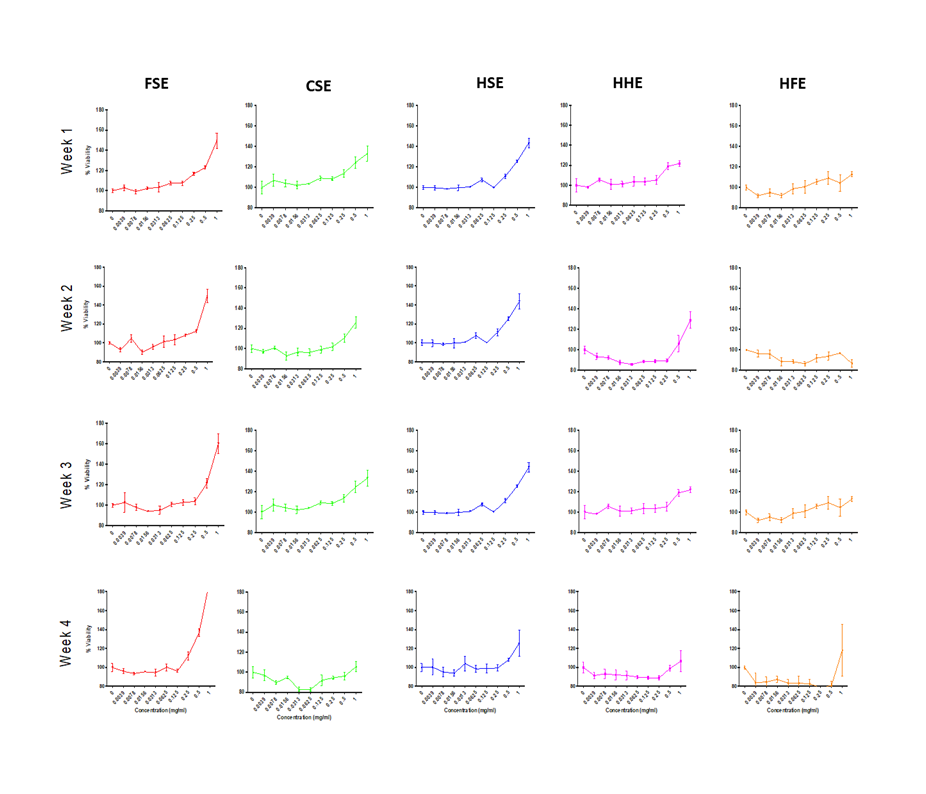

Supplement: Supplemental Information 3 — Treatments were performed in triplicate per concentration and experiments were performed on four different passages of cells over 4 separate weeks. [file peerj-09-12525-s003.png]
